# Immunosensor Incorporating Anti-His (C-term) IgG F(ab’) Fragments Attached to Gold Nanorods for Detection of His-Tagged Proteins in Culture Medium

**DOI:** 10.3390/s100605409

**Published:** 2010-06-01

**Authors:** Michal Wąsowicz, Małgorzata Milner, Dorota Radecka, Krystyna Grzelak, Hanna Radecka

**Affiliations:** 1 Institute of Animal Reproduction and Food Research, Polish Academy of Sciences, Tuwima 10, 10-747 Olsztyn, Poland; E-Mail: m.wasowicz@pan.olsztyn.pl; 2 Institute of Biochemistry and Biophysics, Polish Academy of Sciences, Pawińskiego 5a, 02-106 Warsaw, Poland; E-Mails: gosiam@ibb.waw.pl (M.M.); dorota.radecka@student.uw.edu.pl (D.R.);kg@ibb.waw.pl (K.G.)

**Keywords:** His-tag, F(ab’), gold electrodes, nanorods

## Abstract

Immunosensors based on gold electrodes (electrochemical) or gold discs (optical) modified with 1,6-hexanedithiol, gold nanorods and Anti-His (C-term) monoclonal antibody F(ab’) fragment are described. The antigen detected by the sensing platform is a recombinant histidine-tagged silk proteinase inhibitor (rSPI2-His_6_). Electrochemical impedance spectroscopy (EIS) and surface plasmon resonance (SPR) techniques were used as methods for detection of the antigen. This approach allows to detect the antigen protein in concentration of 10 pg per mL (0.13 pM) of culture medium. The immunosensor shows good reproducibility due to covalent immobilization of F(ab’) fragments to gold nanorods layer.

## 1. Introduction

Histidine-tagged recombinant proteins are widely used in industry and research. The *Pichia* system allowing the production of heterologous proteins is among the most popular [1–3]. Most often a polyhistidine-tag consists of six histidine residues at the C- or N-terminus of the proteins (His_6_-tag). It facilitates the detection and purification of proteins. The tag is poorly immunogenic and generally does not affect the secretion, compartmentalization or folding of the fusion protein within the cell. In most instances, the His-tag does not interfere with the function of proteins as demonstrated for a wide variety of proteins, including enzymes, transcription factors and vaccines [4,5]. Developing a fast, easy and cost-effective detection method of His-tagged proteins would allow for efficient screening of biotechnological processes of proteins production. Currently protein assay relies mostly on popular immunodetection systems including ELISA and Western blot techniques [6]. These are relatively time-consuming and require costly reagents compared to biosensor approach. Immunosensors are a promising alternative to currently used detection systems [7–10]. They are analytical devices comprised of antibodies or their fragments coupled to a transducer and able to generate analytical response related to analyte concentration in a sample. Their ease of use and no need for expensive reagents required for the assay make them an optimal detection system for many purposes [11–14].

Improving immunosensor longevity and selectivity in complex matrices is still a subject of ongoing research. One of the most crucial issues of immunosensor fabrication is associated with the loss of biological activity upon immobilization of antibodies, because of their random orientation on support surfaces [7–10].

In general, the immunoglobulin molecule consists of two polypetide chains F(ab’)_2_ responsible for antigen binding, and an Fc domain, which is not involved in these interaction. The Fc could be removed by enzyme digestions [15,16]. The prepared F(ab’)_2_ or F(ab’) fragments could be self assembled on the gold surface or other functionalized supports due to disulfide or thiol group from the hinge region of immunoglobulin G [17–22].

The immunosensor fabrication process proposed here is shown in Scheme S1 (Supporting Information). The gold nanorods (GNR) have been applied for the underlayer of the immunosensor because of their excellent electron conductivity (EIS measurements) and optical properties (SPR measurements). Gold nanorods are interesting for use in biosensor fabrication thanks to their more suitable properties compared to spherical nanoparticles such as gold colloid.

The end facets of anisotropic Au nanorods are dominated by {111} planes and the side facets by {100} and {110} planes. It was reported that thiol derivatives preferentially bind to the {111} planes of Au nanorods [23–25]. This specific interactions allow Au nanorods assembly perpendicular towards the gold support with using dithiols as the linkers. In contrast, assembling of spherical isotropic Au nanoparticles create ordered 2–D and 3D structures, which are less suitable for selective binding of molecules on the surface [23–25]. The assembling of GNRs onto dithiol SAM deposited on the Au support create well ordered conductive layer with {111} planes on the surface, which is very suitable for oriented covalent immobilization of receptor through Au-S bonding. So, utilizing GNRs in biosensor designing is more efficient compare to using nanoparticles with spherical structures [26].

The study presented concerns the selective binding of antigen rSPI2-His6 present in the sample solution by F(ab’) fragment of antibody immobilized on a surface of the electrode was observed using electrochemical impedance spectroscopy (EIS) as well as surface plasmon resonance (SPR).

## 2. Experimental Section

### 2.1. Chemicals

Alumina 0.3 and 0.05 μm was purchased from Buehler (USA). 1,6-Hexanedithiol (1,6-HDT), l-glycine (Gly), sodium azide (NaN_3_), potassium ferro- and ferricyanides, cetyltrimethylammonium bromide, tetraoctylammonium bromide, gold (III) chloride (HAuCl_4_), and PBS buffer components (NaCl, KCl, Na_2_HPO_4_, KH_2_PO_4_) were purchased from Sigma-Aldrich (Germany). Sulphuric acid, hydrochloric acid, silver nitrate, ethanol, cyclohexane, acetone, and methanol were purchased from POCh (Poland). Anti-His (C-term) monoclonal antibody and bovine serum albumin (BSA) was purchased from Invitrogen Life Technologies (Germany). All aqueous solutions were prepared using deionised water, resistivity 18.2 MΩcm (Millipore). Reagents and solvents were of analytical purity and used without any purification steps. Experiments were carried out at room temperature unless stated otherwise.

### 2.2. Preparation of F(ab’) fragments

According to the manufacturer’s instructions a portion of papain agarose from papaya latex (Sigma) was suspended in 400 μL of H_2_O and incubated at 4 °C for 2 hours, stirring every 15 minutes. Subsequently, the suspension was centrifuged for 5 min (20 °C, 600 g). Then the papain agarose was rinsed twice in the activation buffer (50 mM Na_2_HPO_4_, 20 mM l-cysteine, 1 mM EDTA, pH 7) and activated in the same buffer at 37 °C for 20 min with shaking. Further the papain agarose was washed three times with 3 × digestion buffer (150 mM Na_2_HPO_4_, 3 mM EDTA, pH 7.0), and suspended in 2 × digesting buffer (100 mM Na_2_HPO_4_, 2 mM EDTA, pH 7.0) to the final concentration of 1 μg/μL. This pre-activated papain agarose was used to prepare F(ab’) fragments from Anti-His(C-term) antibody (Invitrogen). IgG (6 μg) was digested in 1x digestion buffer (50 mM Na_2_HPO_4_, 1 mM EDTA, pH 7.0; 20 μL) at 37 °C for 1 hour with constant shaking. The ratio of papain agarose to IgG was about 1:2 (w/w). The digestion was finished by centrifugation (5 minutes, 4 °C, 1,000 g) and the supernatant was used for polyacrylamide gel analysis (Figure S1) and preparation of immunosensor.

### 2.3. Preparation of antigens (rSPI2 proteins)

SPI2 cDNA was cloned into the pPICZαB plasmid carrying the sequences encoding of myc epitope and polyhistidine tag. Transformation of *Pichia pastoris* cells (SMD 1168) by the pPICZαB/SPI2 plasmid and the production of rSPI2-His_6_ protein was done as described previously [26, 27]. Four days after induction of rSPI2-His_6_ expression, the culture medium was collected by centrifugation. rSPI2-His_6_ was purified, from the culture medium, on Ni-NTA-agarose with >95% purity following the procedure described by Kłudkiewicz *et al.* [27]. The culture medium without expressed protein (*Pichia* cells transformed with the pPICZαB plasmid) was used as a control medium. The recombinant SPI2 protein without histidine-tag (rSPI2) was obtained according to the procedure described previously [26,27].

### 2.4. Fabrication of gold nanaorods (GNR)

Gold nanorods were fabricated according to a reported procedure [28]. For gold-seed production, 0.5 mL of 0.01 M HAuCl_4_ trihydrate solution in water and 0.5 mL of 0.01 M sodium citrate solution in water were added to 18 mL of deionized water and stirred. Next, 0.5 mL of freshly prepared 0.1 M NaBH_4_ was added and the solution color changed from colorless to orange. Stirring was stopped and the solution was left undisturbed for 2 hours. The resulting spherical nanoparticles were used as gold seed. For gold nanorod growth from the seeds, three flasks were labeled A, B and C. Growth solutions A and B consisted of 9 mL of 0.1 M cetyltrimethylammonium bromide (CTAB) in water, 0.25 mL of 0.01 M HAuCl_4_ trihydrate in water, 50 μL of 0.1 M ascorbic acid. Growth solution C consisted of 90 mL 0.1 M CTAB, 2.5 mL 0.01 M HAuCl_4_, 0.5 mL 0.1 M ascorbic acid. The seed solution was added to growth solution A and shaken for 3 seconds. Then it was poured into solution B, shaken for 4 seconds and added to solution C. The mixture turned reddish in several minutes and was left overnight at room temperature. Excess CTAB was removed by centrifugation (Eppendorf MiniSpin, 1,000 rpm 5 min). Supernatant was removed and the pellet resuspended in water and stored in +4 °C.

### 2.5. Characterization of gold nanorods (GNR) with UV-Vis and Atomic Force Microscopy (AFM)

The suspension of gold nanorods obtained according to the procedure described in Paragraph 2.4 was characterized using UV-Vis spectrophotometry and Atomic Force Microscopy Quesant-Scope Universal Scanning (Ambios Technology, USA). Scans were performed in intermittent contact, broadband wavemode using NSC16 (W_2_C, Si_3_N_4_) tips oscillating at base frequency of 170 kHz. Tip curvature radius was about 10 nm. Dimensions of scanned objects were measured using Quesant software delivered with the microscope.

### 2.6. Preparation of immunosensor

Gold electrodes were cleaned in alumina with 0.3 μm and 0.05 μm particle diameter size, 5 minutes each). The alumina suspensions were spotted on the BAS microcloth polishing pad and gold electrodes were gently polished by doing small circles in the one direction during 5 minutes. Afterwards they were carefully rinsed and sonicated in Milli-Q water for 30 seconds. Electrochemical cleaning electrode setup: Au—working, Pt—counter, Ag/AgCl—reference. Conditions: 0.5 M H_2_SO_4_ water solution, minimum potential—0.3 V, maximum potential 1.5 V, scan rate 100 mV/s, number of cycles: 3, 20 and 5. Clean gold electrodes were rinsed with ethanol and put in 10 mM 1,6-HDT in ethanol solution for 18 hours at room temperature. Test tubes containing electrodes and 1,6-HDT solution were sealed with Teflon tape and Parafilm to avoid solvent evaporation. Subsequently electrodes were rinsed with ethanol and water respectively. Next, electrodes were put in test tubes upside down and a 10 μL droplet of gold nanorods (GNR) suspension obtained using the procedure described in paragraph 2.4. was spotted on each gold surface. The test tubes were sealed with Parafilm and put in +4 °C for 18 hours. Next, electrodes were rinsed with water and PBS pH 7.4 (137 mM NaCl, 10 mM phosphate, 2.7 mM KCl). Next, 10 μL droplets of F(ab’) solution (0.3 μg/μL) were put on the surface of each electrode and kept in +4 °C for 20 hours in sealed test tubes. After incubation, electrodes were carefully rinsed with PBS. BSA solution (in 0.1 M PBS pH 7.4) in concentration of 0.5% (m/v) was used for blocking of unspecific binding sites. A 10 μL droplet was spotted on each electrode and incubated for 2 hours. Finally, electrodes were rinsed with PBS. Fully modified electrodes were stored in refrigerator (+ 4 °C) in PBS buffer until use.

### 2.7. Electrochemical impedance spectroscopy (EIS) and cyclic voltammetry (CV) measurements

EIS and CV measurements were performed using Autolab potentiostat-galvanostat (Eco Chemie, The Netherlands). Electrochemical cell setup: Au (BAS, USA) as the working electrode, Ag/AgCl as reference electrode, Pt as counter electrode. Measurements were performed in solution comprised of 0.1 M PBS pH 7.4 and K_3_[Fe(CN)_6_]/K_4_[Fe(CN)_6_] (0.5 mM each). EIS setup: frequency range 10 kHz to 100 mHz, formal potential 0.170 V. Electron transfer resistance (*R_i_*) was calculated from spectra fitted by Autolab software and electrode responses were expressed as: (*R_i_* − *R*_0_)/*R*_0_ where *R_i_* is the electron transfer resistance measured in the presence of the analyte and *R*_0_ the electron transfer resistance without the analyte. The amount of sample used was 10 μL (a drop spotted on gold electrode surface). Incubation time at room temperature was 20 minutes in a sealed test tube. After applying the sample, the electrodes were conditioned in 0.1 M PBS pH 7.4 for 5 minutes. Concentrations of sample solutions of rSPI2-His_6_ protein were as follows: 10 pg/mL (0.13 pM), 30 pg/mL (0.39 pM), 100 pg/mL (1.31 pM), 300 pg/mL (3.93 pM) and 1 ng/mL (13.10 pM). The concentrations of rSPI2 were as follow: 10 pg/mL (0.15 pM), 30 pg/mL (0.44 pM), 100 pg/mL (1.47 pM), 300 pg/mL (4.41 pM) and 1 ng/mL (14.70 pM). After the EIS assay the electrodes were put in 0.2 M Gly-HCl pH 2.8 buffer 3 × 2 min and rinsed with 0.1 M PBS pH 7.4. Regenerated electrodes were used for further EIS assays until reaching 10th cycle, after which they were cleaned and fresh modification was applied.

### 2.8. Surface Plasmon Resonance (SPR)

An Autolab SPRINGLE ver. 4.2. system from Eco Chemie B. V. (The Netherlands) apparatus was used. SPR gold discs were cleaned in piranha solution (H_2_SO_4_ 96% and H_2_O_2_ 30% 7:3) 3 × 1 min and thoroughly washed in Milli-Q water. After rinsing with ethanol, gold discs were put in 10 mM 1,6-HDT in ethanol for 18 hours at room temperature. Next, discs were rinsed with ethanol and water, and were covered with 50 μL gold nanorods solution for 18 hours at +4 °C. Next, 50 μL of F(ab’) solution (0.3 μg/μL) were put on the disc surface and kept in +4 °C for 20 hours in sealed glass containers. After incubation, discs were carefully rinsed with PBS. Next, F(ab’) modified gold discs were put in 5 mL of 0.05% BSA solution at +4 °C for 2 hours. Finally the discs were rinsed and put in PBS buffer for storing between experiments.

Upon obtaining a stable reading (angle shift not greater than 0.1 milidegrees per minute) in PBS buffer, 50 μL of the first sample solution of analyte, rSPI2-His_6_ or rSPI2, was placed on the F(ab’) modified gold disc. After 20 minutes the sample solution was rinsed off with PBS buffer. The stable value of angle shift was recorded after 200 s. Samples of rSPI2-His_6_ in concentrations 10 pg/mL (0.13 pM), 30 pg/mL (0.39 pM), 100 pg/mL (1.31 pM), 300 pg/mL (3.93 pM) and 1 ng/mL (13.10 pM) were used. The concentrations of rSPI2 were as follow: 10 pg/mL (0.15 pM), 30 pg/mL (0.44 pM), 100 pg/mL (1.47 pM), 300 pg/mL (4.41 pM) and 1 ng/mL (14.70 pM). After the SPR assay the modified gold discs were put in 0.2 M Gly-HCl pH 2.8 buffer 3 × 2 min and rinsed with 0.1 M PBS pH 7.4. The regenerated gold discs were used for further SPR assays until reaching 10th cycle, after which they were cleaned and fresh modification was applied.

## 3. Results and Discussion

### 3.1. Characterization of gold nanorods (GNR)

**Figure 1.**
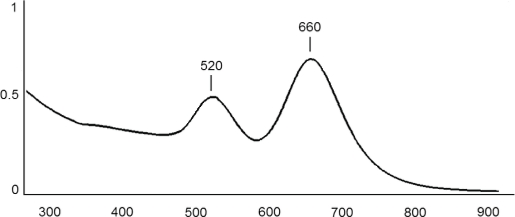
The typical UV-Vis spectrum of GNR suspension obtained according to procedure described in Paragraph 2.4.

A typical UV-Vis spectrum of GNR suspension is shown in Figure 1. Absorption peaks at 520 nm and 660 nm correspond to the transverse and longtitudinal plasmon bands, respectively. The positions of the peaks are influenced by the nanoparticle aspect ratio. Research done by Toderas *et al.* [29] confirm that such spectrum can be obtained for nanorods of reported size and aspect ratio.

**Figure 2.**
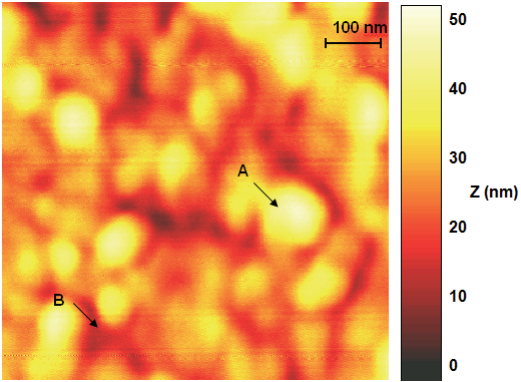
AFM image of gold nanorods layer formed on 1,6-HDT modified gold plate. A—protruding nanorod, B—cracks between nanorods.

A typical nanorod AFM image is shown in Figure 2. The nanorods are perpendicular to the hexane-dithiol SAM. It is clearly visible that GNRs create well ordered layers with a surface larger than the Au support and more suitable for immobilization of antibodies. Results obtained using AFM show that nanorods used in this study were 70 to 90 nm wide (W = 80 nm, SD = 14 nm, n = 50). This dimension can be measured on a formed nanorod layer, being the only visible full dimension. The UV-Vis spectrum of GNR (Figure 1) confirmed the aspect ratio of 2.5 [29]. This means the average nanorod in our study was 80 nm wide and 200 nm long.

The use of nanorods as an underlayer for electrochemical biosensors is justified by their properties. Layers formed by gold nanorods are highly ordered, with nanorods protruding in one direction. This might help prevent local inconsistencies of the layer that may influence repeatability of the layer formation. In addition the deposition of GNR on the Au support increases the electrochemically available surface in comparison to bare gold or even gold nanospheres (Figure 2).

### 3.2. Characterization of the electrochemical immunosensor

The process of immunosensor fabrication is shown in Scheme S1 (Supporting Information). F(ab’) fragments of immunoglobulins, which were used in the present study, are proteins with molecular mass of ca. 48 kDa. They were obtained by antibody digestion with low concentration of papain [15,16] (Scheme S2, Figure S1, Supporting Information).

**Figure 3.**
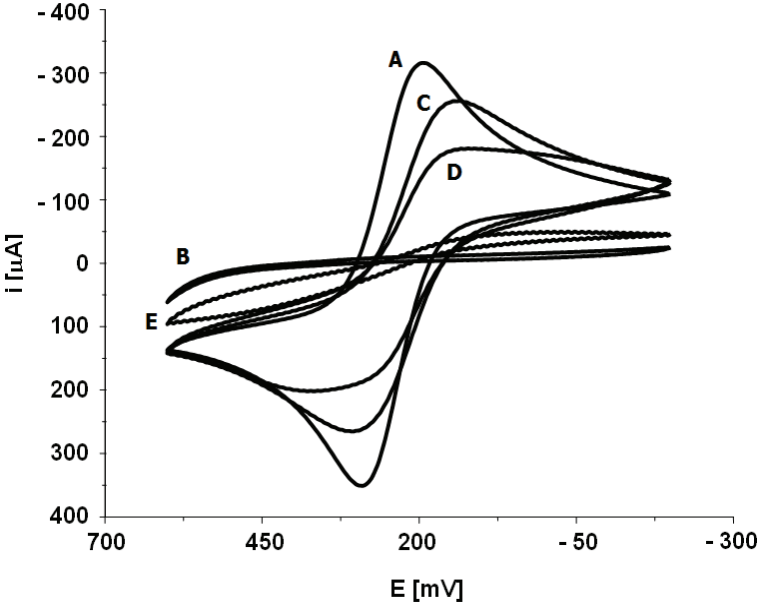
Cyclic voltammograms of (A) clean gold electrode surface; (B) 1,6-hexanedithiol/Au electrode; (C) gold nanorods/1,6-hexanedithiol/Au electrode; (D) F(ab’)/gold nanorods/1,6-hexanedithiol/Au electrode; (E) bovine serum albumin/F(ab’)/gold nanorods/1,6-hexanedithiol/Au electrode. Electrochemical cell setup: Au (BAS, USA) as the working electrode, Ag/AgCl as reference electrode, Pt as counter electrode. Solution composition: of 0.1 M PBS pH 7.4 and K_3_[Fe(CN)_6_]/K_4_[Fe(CN)_6_] (0.5 mM each).

**Figure 4.**
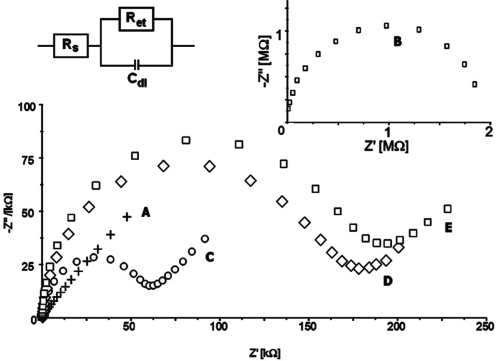
Impedance spectra of (A) clean gold electrode surface; (B) 1,6-hexanedithiol/Au electrode; (C) gold nanorods/1,6-hexanedithiol/Au electrode; (D) F(ab’)/gold nanorods/1,6-hexanedithiol/Au electrode; (E) bovine serum albumin/F(ab’)/gold nanorods/1,6-hexane-dithiol/Au electrode. Circuit model used for fitting Nyquist plots in inset. R_s_—solution resistance, R_et_—electron transfer resistance, C_dl_—double layer capacitance. Electrochemical cell setup: Au (BAS, USA) as the working electrode, Ag/AgCl as reference electrode, Pt as counter electrode. EIS setup: frequency range 10 kHz to 100 mHz, formal potential 0.170 V. Solution composition: of 0.1 M PBS pH 7.4 and K_3_[Fe(CN)_6_]/K_4_[Fe(CN)_6_] (0.5 mM each).

All modification steps were controlled using CV (Figure 3) and EIS (Figure 4) in the presence of 1 mM K_3_Fe(CN)_6_/K_4_Fe(CN)_6_ (1:1) as redox marker. Bare gold electrodes have no obstacles affecting electron transfer which results in peak separation 66 ± 2 mV. EIS control of this step produces an almost straight line as Nyquist plot (R_et_ = 100 ± 10 kΩ). This indicates a diffusion controlled electrochemical process, not limited by electron transfer.

The first modification step includes coating the gold layer with 1,6-hexanedithiol forming a self-assembled monolayer. This results in blocking the surface for the redox marker and can be seen in a cyclovoltammogram with no apparent peaks. Nyquist plots obtained by EIS measurements show a large semicircle at higher frequencies indicating an electron transfer limited electrode process. This increase the electron transfer resistance significantly (R_et_ = 730 ± 30 kΩ). Next the gold nanorods layer is formed to provide a conductive flexible underlayer for biomolecules [26,30–32]. The immobilization of gold nanorods (GNR) on the 1,6-hexanedithiol SAM decrease electron transfer resistance. The redox peaks separation in the range 82 ± 3 mV were observed. EIS control of this step confirmed this phenomenon. For gold GNR layer deposited onto 1,6-hexanedithiol SAM electron transfer resistances R_et_ = 180 ± 15 kΩ were recorded. F(ab’) fragments were bound covalently to the GNR layer through Au-S covalent bond. This provides both the right orientation of the receptors as well as strong binding to the supporting layer [17–22,33]. F(ab’) fragments create the insulating layer on the electrode surface. This results in an increase of the CV peak separation to 130 ± 5 mV and an increase of the electron transfer resistance in EIS (R_et_ = 320 ± 22 kΩ). Blocking remaining sites of gold nanorods layer with BSA almost totally closed the electrode for the redox marker. Calculated R_et_ increased to 650 ± 25 kΩ.

### 3.3. Determination of the rSPI2-His_6_ protein by EIS measurements

**Figure 5.**
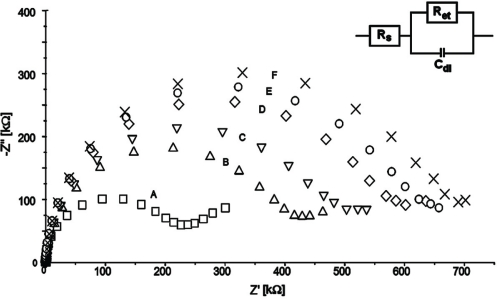
Impedance spectra of immunosensor: in pure PBS (A); in the presence of antigen protein rSPI2-His6 in concentrations 10 pg/mL (0.13 pM) (B); 30 pg/mL (0.39 pM) (C); 0.1 ng/mL (1.31 pM) (D); 0.3 ng/mL (3.93 pM) (E) 1.0 ng/mL (13.10 pM) (F). Electrochemical cell setup: modified Au (BAS, USA) as the working electrode, Ag/AgCl as reference electrode, Pt as counter electrode. EIS setup: frequency range 10 kHz to 100 mHz, formal potential 0.170 V. Solution composition: of 0.1 M PBS pH 7.4 and K_3_[Fe(CN)_6_]/K_4_[Fe(CN)_6_] (0.5 mM each). Circuit model used for fitting Nyquist plots in inset. R_s_—solution resistance, R_et_—electron transfer resistance, C_dl_—double layer capacitance.

A typical response of the immunosensor measured by EIS is shown in Figure 5. Electron transfer resistance measured for the immunosensor in pure PBS buffer R_0_ (Figure 5. curve A) is used to calculate relative response towards a specific analyte. Addition of the specific antigen increases the electron transfer resistance *R_i_* (Figure 5. curves B–F).

Electrochemical impedance measurements are characterized by relatively short assay times (4 minutes per concentration). Results obtained were well reproducible (standard deviations from nine repetitions did not exceed 8% of response values). The classic gold electrodes could be replaced with micro size electrodes. This makes EIS technique a potential candidate for development of miniaturized sensors. The proposed immunosensor was very selective and sensitive. Table 1 shows comparison of results obtained in the presence of His–tagged antigen and those obtained in the presence of antigen without his–tag. The values in Table 1 are linear equation coefficients obtained by putting concentration on logarithmic X axis and average calculated analytical response on Y axis. The first equation in Table 1 corresponds to an array of concentrations of the specific antigen (rSPI2-His_6_) in pure buffer. This assay, as expected, yielded the highest sensitivity and repeatability. It was performed to characterize the sensor in optimal conditions. The second equation corresponds to the negative control (rSPI2 protein without his_6_ -tag) in 0.1 M PBS pH 7.4 buffer. It is characterized by low slope and relatively high noise, which is expected from a negative control. Third set of data shows the performance of the immunosensor in matrix—*Pichia* culture medium. It is comparable to the positive control (rSPI2-His_6_ in PBS) in optimal conditions. Slope at the level of 83.6% of the initial value shows that the loss of sensitivity when used in matrix is not much a concern. The last result is the response of the sensor towards the matrix alone.

The immunosensor incorporating GNR and anti-His IgG F(ab’) fragments allows for detecting 10 pg/mL (130 fM) of rSPI2-His_6_ protein in PBS buffer or in *Pichia* culture medium in the same range of concentrations. The linear analytical response ranged from 10 pg/mL (130 fM) to 1 ng/mL (13.10 pM).

Reusability is very important sensor parameter. Among the buffers used for regeneration of immunosenors after performing the detection of rSPI2-His_6_ antigen, 0.1 M glycine-HCl pH 2.8 buffer was the most efficient. Nevertheless, after regeneration ten times in 0.1 M glycine-HCl pH 2.8 buffer, the immunosensor displayed sensitivity towards rSPI2-His_6_ protein with linear response slope of 52% the initial (compared to freshly prepared sensor) value. In the case of an immunosenor incorporating whole antibody deposited on the gold nanoparticles via electrostatic interactions, a 50% decrease of sensitivity towards rSPI2-His_6_ protein was observed after the 4th regeneration in 0.1 M glycine-HCl pH 2.8 buffer [26]. Thus, it was shown that the covalent immobilization of F(ab’) fragments on the GNR improved the reusability of immunosensor.

Based on the parameters such as very low detection limit, 10 pg of antigen per one mL, negligible influence of culture media, reusability, proposed immunosensor could compete with the immunosensors incorporating whole antibody and based on electrochemical impedance techniques already reported in the literature [34–37]. The main disadvantage of proposed EIS based immunosensors include problematic calibration, which is done in a relative manner. It means that the basic value of impedance can vary among individual sensors and it must be compared with the values obtained for studied samples to yield an analytical response. On the other hand SPR equipment is still relatively expensive compared to electrochemical hardware.

### 3.4. Determination of the rSPI2-His_6_ protein by SPR measurements

**Figure 6.**
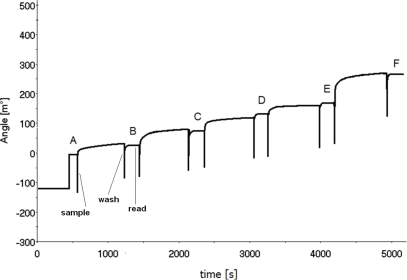
Real time response of the immunosensor measured by SPR: in PBS (**A**); in the presence of antigen protein rSPI2-His_6_ in concentrations:10 pg/mL (0.13 pM), 30 pg/mL (0.39 pM), 100 pg/mL (1.31 pM), 300 pg/mL (3.93 pM) and 1 ng/mL (13.10 pM). Solution composition: rSPI2-His_6_ protein in PBS pH 7.4 (137 mM NaCl, 10 mM phosphate, 2.7 mM KCl).

A typical immunosensor response to the specific antigen recorded using the SPR equipment is shown in Figure 6. The first plateau is the basic level of angle shift observed in pure PBS buffer. Successive plateaus correspond to increasing concentrations of the rSPI2-His_6_ protein. Their angle shift values were used for calculations of parameters characterizing the immunosensor response and are summarized in Tables 3 and 4.

Concentration range of rSPI2-His_6_: 10 pg/mL (0.13 pM), 30 pg/mL (0.39 pM), 100 pg/mL (1.31 pM), 300 pg/mL (3.93 pM) and 1 ng/mL (13.10 pM) and incubation time (20 min at room temperature) were analogous to those used for EIS. The calibration curve slope for the full concentration range in the case of SPR is about four times higher than in the case of EIS. Limit of detection, linear response range and capacity for regeneration are similar for both methods. Repeatability of the results for the immunosensor both in case of EIS and SPR is good. Standard deviations are relatively low (ranging from 1.1 to 2.4, see Tables 1–4). Noise to signal ratio is very good, 2% for EIS and 5% for SPR. Also the sensitivity loss when used in matrix is very satisfactory (88.7% of initial signal retained in matrix).

The measurement for full concentration range can be performed in ca. 2 hours and does not require costly labels. The SPR equipment, being more complicated and expensive than a potentiostat, is not yet suitable for miniaturized biosensors, however the sensitivity and stability of SPR measurements are impressive.

GNR proved to be an optimal underlayer for an optical sensing platform. GNR naturally enhance SPR readings because plasmons appearing on their surface are easily affected by insulators (in this case antigen proteins) adsorbed [14,38]. Regeneration results shown in Table 4 proved that reusability of the SPR immunosensor is much comparable to this of EIS immunosensor. After a week of regeneration/assay cycles formerly good sensitivity begins to drop noticeably. The several examples of SPR immunosensors incorporating F(ab’) or F(ab’)_2_ fragments of antibody have been already reported [17–19,22]. Their main drawback is rather complex and multistep preparation of sensing platform and sensing element immobilization. The way of fabrication of SPR immunosensor proposed is simpler and less chemical consuming. The application of GNRs provide not only good conductive underlayer, but also allows for one step covalent immobilization of F(ab’) fragment. Because of that the immunosensor proposed displayed very good analytical parameters such as low detection limit 10 pg/mL, lack of the influence of the complex medium matrix and reproducibility (standard deviations from 9 repetitions did not exceed 8% of response values).

The immunosensor incorporating F(ab’) fragments covalently attached through Au-S bonds on the surfaces of gold nanoparticles showed better reusability in the comparison with one incorporating whole IgG antibody [26]. This is mainly because of different immobilization strategy. The covalent immobilization is stronger in comparison to electrostatic interactions between antibody and sensing platform. In addition, covalent bonds between Au atoms of GNPs and SH group of F(ab’) fragments enabling the appropriate orientation for antigen binding [39].

## 4. Conclusions

Immunosensor incorporating Anti-His IgG F(ab’) fragment and gold nanorods for detection of his-tagged proteins is able to detect 10 pg/mL (0.13 pM) of antigen. The recognition process take place on the surface, not in the solution. Therefore, the necessary amount of F(ab’) fragments is very small (6 × 10^−11^ mols of F(ab’) per electrode). It also requires no labels, which is a distinct advantage. These traits make it an easy to use and relatively cheap method of detecting antigens in complex medium. The limit of detection remains almost the same in culture medium as was observed in PBS buffer. Analytical response range for the immunosensor proposed is between 10 pg/mL (0.13 pM) and 1 ng/mL 13.1 pM) which is better in the comparison of other already reported. Immunosensor incorporating F(ab’) fragments is more reusable than one incorporating whole antibody IgG.

## Figures and Tables

**Table 1.** EIS responses of the immunosensor incorporating Anti-His IgG F(ab’) fragment towards rSPI-His_6_ in PBS buffer pH 7.4 and in the presence of *Pichia pastoris* culture medium. Control samples: protein rSPI2 in PBS and medium alone.

**Analyte**	**Response**
rSPI2-His_6_ in PBS	y = **9.58** log(x) + 49.21 r^2^ = 0.997, σ =1.5
rSPI2 in PBS	y = **0.21** log(x) + 2.35 r^2^ = 0.659, σ =1.9
rSPI2-His_6_ in culture medium	y = **8.01** log(x) + 43.24 r^2^ = 0.998, σ =1.1
Culture medium without rSPI2 or rSPI2-His_6_	y = **0.87** log(x) + 3.98 r^2^ = 0.989, σ =1.2

y = EIS response ([(*R_i_* − *R_0_*)/*R_0_*]; *R_i_*—electron transfer resistance at the presence of analyte; *R_0_*—electron transfer resistance in the absence of analyte); x = analyte concentration; 10 pg/mL < X < 1ng/mL rSPI2-His_6_; r^2^—correlation coefficient; σ—standard deviation, n = 9

**Table 2.** Regeneration of the immunosensor incorporating Anti-His IgG F(ab’) fragment regenerated with 0.1 M Gly-HCl pH 2.8 buffer. Response measured by EIS towards rSPI2-His_6_ in PBS buffer pH 7.4.

rSPI2-His_6_ in PBS	y = **9.58** log(x) + 49.21 r^2^ = 0.997, σ =1.5
Response after regeneration—Cycle 1	y = **9.54** log(x) + 48.94 r^2^ = 0.998, σ =1.5
Cycle 2	y = **9.50** log(x) + 48.63 r^2^= 0.997, σ =1.8
Cycle 3	y = **9.48** log(x) + 48.67 r^2^ = 0.999, σ =1.6
Cycle 4	y = **9.36** log(x) + 44.84 r^2^ = 0.998, σ =1.9
Cycle 5	y = **9.21** log(x) + 40.08 r^2^ = 0.994, σ =1.3
Cycle 6	y = **9.03** log(x) + 37.14 r^2^ = 0.991, σ =1.5
Cycle 7	y = **7.76** log(x) + 32.68 r^2^ = 0.992, σ =1.7
Cycle 8	y = **5.49** log(x) + 24.70 r^2^ = 0.995, σ =1.2
Cycle 9	y = **4.38** log(x) + 17.02 r^2^ = 0.993, σ =1.7

y = EIS response ([(*R_i_* − *R*_0_)/*R*_0_]; *R_i_*—electron transfer resistance at the presence of analyte; *R_0_*—electron transfer resistance in the absence of analyte); x = analyte concentration; 10 pg/mL < X < 1ng/mL rSPI2-His_6_; r^2^—correlation coefficient; σ—standard deviation, n = 9

**Table 3.** SPR responses of the immunosensor incorporating Anti-His IgG F(ab’) fragment towards rSPI-His_6_ in PBS buffer pH 7.4 (137 mM NaCl, 10 mM phosphate, 2.7 mM KCl) and in the presence of *Pichia pastoris* culture medium. The control sample: protein rSPI2 in PBS and medium alone.

**Analyte**	**Response**
rSPI2-His_6_ in PBS	y = **36.40** log(x) + 183.20 r^2^ = 0.987, σ = 2.3
rSPI2 in PBS	y = **1.92** log(x) + 13.54 r^2^ = 0.834, σ = 1.9
rSPI2-His_6_ in culture medium	y = **32.28** log(x) + 145.29 r^2^ = 0.979, σ = 2.4
Culture medium without rSPI2 or rSPI2-His_6_	y = **3.05** log(x) + 10.74 r^2^= 0.963, σ = 2.0

y = SPR response (milidegrees); x = analyte concentration; 10 pg/mL < X < 1ng/mL rSPI2-His_6_; r^2^—correlation coefficient; σ—standard deviation, n=9

**Table 4.** Regeneration of the immunosensor incorporating Anti-His IgG F(ab’) fragment regenerated with 0.1 M Gly-HCl pH 2.8 buffer. Response measured by SPR towards rSPI2-His_6_ in PBS buffer pH 7.4.

rSPI2-His_6_ in PBS	y = **36.40** log(x) + 183.2 r^2^ = 0.987, σ = 2.3
Response after regeneration—Cycle 1	y = **35.62** log(x) + 176.9 r^2^ = 0.952, σ = 2.2
Cycle 2	y = **31.81** log(x) + 153.3 r^2^= 0.956, σ = 2.4
Cycle 3	y = **30.50** log(x) + 146.1 r^2^= 0.975, σ = 1.9
Cycle 4	y = **29.35** log(x) + 140.7 r^2^ = 0.991, σ = 2.0
Cycle 5	y = **28.38** log(x) + 137.5 r^2^ = 0.994, σ = 1.6
Cycle 6	y = **26.87** log(x) + 125.9 r^2^ = 0.986, σ = 1.8
Cycle 7	y = **24.28** log(x) + 118.3 r^2^ = 0.992, σ = 1.7
Cycle 8	y = **20.06** log(x) + 112.6 r^2^ = 0.995, σ = 1.5
Cycle 9	y = **18.97** log(x) + 104.7 r^2^ = 0.993, σ = 1.6

y = SPR response (milidegrees); x = analyte concentration; 10 pg/mL < X < 1ng/mL rSPI2-His_6_; r^2^—correlation coefficient; σ—standard deviation, n = 9
